# A coupling strategy for a first 3D-1D model of the cardiovascular system to study the effects of pulse wave propagation on cardiac function

**DOI:** 10.1007/s00466-022-02206-6

**Published:** 2022-07-09

**Authors:** Federica Caforio, Christoph M. Augustin, Jordi Alastruey, Matthias A. F. Gsell, Gernot Plank

**Affiliations:** 1 Institute of Mathematics and Scientific Computing, NAWI Graz, University of Graz, Graz, Austria; 2 Gottfried Schatz Research Center: Division of Biophysics, Medical University of Graz, Graz, Austria; 3 BioTechMed-Graz, Graz, Austria; 4 Department of Biomedical Engineering, Division of Imaging Sciences and Biomedical Engineering, King’s College London, King’s Health Partners, St. Thomas’ Hospital, London, SE1 7EH UK

**Keywords:** 3D-1D coupling, Multiphysics modelling, Cardiovascular modelling, Cardiac electromechanics, Pulse wave propagation

## Abstract

A key factor governing the mechanical performance of the heart is the bidirectional coupling with the vascular system, where alterations in vascular properties modulate the pulsatile load imposed on the heart. Current models of cardiac electromechanics (EM) use simplified 0D representations of the vascular system when coupling to anatomically accurate 3D EM models is considered. However, these ignore important effects related to pulse wave transmission. Accounting for these effects requires 1D models, but a 3D-1D coupling remains challenging. In this work, we propose a novel, stable strategy to couple a 3D cardiac EM model to a 1D model of blood flow in the largest systemic arteries. For the first time, a personalised coupled 3D-1D model of left ventricle and arterial system is built and used in numerical benchmarks to demonstrate robustness and accuracy of our scheme over a range of time steps. Validation of the coupled model is performed by investigating the coupled system’s physiological response to variations in the arterial system affecting pulse wave propagation, comprising aortic stiffening, aortic stenosis or bifurcations causing wave reflections. Our first 3D-1D coupled model is shown to be efficient and robust, with negligible additional computational costs compared to 3D-0D models. We further demonstrate that the calibrated 3D-1D model produces simulated data that match with clinical data under baseline conditions, and that known physiological responses to alterations in vascular resistance and stiffness are correctly replicated. Thus, using our coupled 3D-1D model will be beneficial in modelling studies investigating wave propagation phenomena.

## Introduction

Computational modelling of cardiac electromechanics (EM) is increasingly proposed and pursued as an effective tool for investigating cardiac physiology and numerous pathological conditions [1, 2]. Computer models have been used for quantitative analysis, since they have the potential to assess valuable diagnostic information and represent a strong predictive tool for analysing the patient-specific response to a given treatment [3]. However, this is a very open and challenging field of research, since cardiac function relies on complex biophysical aspects that are strongly interlinked [4]. Indeed, the muscle contraction is stimulated by electrical activation and strongly interacts with intraventricular blood and the vascular system to transport nutrients and clear waste products. As a consequence, efficient computational tools are required that account for the complex physiology and multiphysics of the heart and can be practically implemented and integrated into the clinic [5].

In this context, numerous mathematical models have been reported in the literature that aim to model the effect of the vascular system on cardiac function. These range from simple lumped zero-dimensional (0D) Windkessel-type models [6–8], to more refined 1D models [9–11]. While 0D models are suitable as global models of afterload, providing a simple way to evaluate global vascular dynamics, they do not consider any effects due to pulse wave transmission and thus are unable to predict important markers such as pulse wave velocity. In contrast, 1D blood flow models enable an efficient representation of the effects of pulse wave propagation and reflection in the circulation. Consequently, 1D models may be preferred over 0D models when local vascular changes or distributed properties, e.g., vessel branching, tapering, stenoses, and their effect on central pressure waveforms are studied and when pulse wave transmission effects are under investigation [12].

To date, most 3D computational models of the heart proposed in the literature consider lumped 0D models as boundary conditions to model the circulation [1, 13–17] for ease of implementation and calibration. To the best of our knowledge, no studies propose an effective and stable method to couple a 3D cardiac model to a 1D description of the circulation, which can provide new insights into the physiology and analyse the effects of disrupted wave propagation on cardiovascular dynamics. This is clinically relevant, since the impact of increased stiffness and pulsatile load on the circulation and cardiac performance have been clinically documented not only for cardiovascular events, but also for left and right ventricle dysfunctions [18].

In order to address this need, in this methodological paper we present a novel *in silico* model based on a multidimensional approach, namely a 3D EM model of cardiac function together with a 1D model of the human circulation. The building blocks of the coupled model and the main aspects of model parameterisation are thoroughly described in the text. The coupling strategy is inspired by recent methods proposed for 3D-0D models [13, 19] and is based on the solution of a saddle-point problem for the volume and pressure in the cardiac cavity. A personalised 3D EM model of the left ventricle (LV) [1] coupled to a 1D vascular model [20] is built for the first time and employed in numerical test cases to demonstrate robustness and stability of the numerical scheme over a range of time steps. The physiological response of the calibrated 3D-1D model is investigated and validated with clinical data under baseline conditions and in different conditions affecting pulse wave transmission, such as aortic stiffening, aortic narrowing and complex vessel networks involving numerous bifurcations causing wave reflections. We show the ability of the coupled 3D-1D model to correctly replicate known physiological behaviours related to alterations in vascular resistance and stiffness. The computational cost of the presented coupled 3D-1D model is comparable to that of standard 3D-0D models.

Due to its efficiency and robustness, our model is particularly suitable for clinical applications where wave transmission effects need to be investigated.

## Methodology

### 3D Electromechanical cardiac model

We first outline the mathematical models to describe the most fundamental aspects of cardiac function, comprising electrophysiology, passive and active mechanics.

#### Electrophysiology

A recently developed Reaction-Eikonal (R-E) model [21] is considered for the generation of electrical activation sequences serving as a trigger for active stress generation in cardiac tissue. This hybrid R-E model combines a standard reaction diffusion model based on the monodomain equation with an eikonal model. In more detail, we consider the eikonal equation given as

1 $$\begin{aligned} \left\{ \begin{array}{rcll} \sqrt{\nabla _{{\mathbf {X}}} t_\mathrm {a}^\top \, {\mathbf {V}} \, \nabla _{{\mathbf {X}}} t_\mathrm {a}} &{} = &{} 1 \qquad &{} \text {in } \Omega _0, \\ t_\mathrm {a} &{} = &{} t_0 &{} \text {on } \Gamma _0^{*}, \end{array} \right. \end{aligned}$$

where $(\nabla _{{\mathbf {X}}})$ is the gradient with respect to the end-diastolic reference configuration $\Omega _{0}$, $t_\mathrm {a}$ is a positive function that describes the wavefront arrival time at location ${\mathbf {X}}\in \Omega _0$, and $t_0$ denote the initial activations at locations $\Gamma _0^*\subseteq \Gamma _{0,\mathrm {N}}$. The symmetric positive definite $3 \times 3$ tensor ${\mathbf {V}}({\mathbf {X}})$ contains the squared velocities $\left( v_{{\mathbf {f}}}({\mathbf {X}}),v_{{\mathbf {s}}}({\mathbf {X}}),v_{{\mathbf {n}}}({\mathbf {X}})\right)$ associated with the tissue’s eigenaxes ${\mathbf {f}}_0$, ${\mathbf {s}}_0$, and ${\mathbf {n}}_0$. Then, the arrival time function $t_\mathrm {a}({\mathbf {X}})$ is used in a modified monodomain R-D model given as

2 $$\begin{aligned} \beta C_\mathrm {m} \frac{\partial V_\mathrm {m}}{\partial t} = \nabla _{{\mathbf {X}}} \cdot \varvec{\sigma }_\mathrm {i} \nabla _{{\mathbf {X}}} V_\mathrm {m} + I_\mathrm {foot} - \beta I_\mathrm {ion}, \end{aligned}$$

where an arrival time dependent foot current, $I_{\mathrm {foot}}(t_\mathrm {a})$, is added, in order to mimic sub-threshold electrotonic currents and produce a physiological foot of the action potential. The key advantage of this R-E model is that it enables to compute activation sequences at much coarser spatial resolutions without being afflicted by the spatial undersampling artefacts leading to conduction slowing or even numerical conduction blocks that are observed in standard R-D models. The tenTusscher–Noble–Noble–Panfilov model of the human ventricular myocyte [22] is employed to model Ventricular electrophysiology.

#### Passive cardiac mechanics

The deformation of the heart is governed by imposed external loads such as pressure in the cavities or from surrounding tissue and active stresses intrinsically generated during contraction. The cardiac tissue is characterised as a hyperelastic, nearly incompressible, anisotropic material with a nonlinear stress-strain relationship. Mechanical deformation is described by Cauchy’s equation of motion leading to a boundary value problem

3 $$\begin{aligned} \rho _0\,\ddot{ {\mathbf {u}}}(t,{\mathbf {X}})-\nabla _{{\mathbf {X}}}\cdot \mathbf {F}\mathbf {S}({\mathbf {u}},{\mathbf {X}}) = {\mathbf {b}}_0({\mathbf {X}}) \ \text{ in } \Omega _0 , \end{aligned}$$

for $t \in [0, T]$, where $\rho _0$ is the density in the Lagrange reference configuration, ${\mathbf {u}}(t,{\mathbf {X}})$ is the unknown nodal displacement, $\dot{{\mathbf {u}}}(t,{\mathbf {X}})$ is the nodal velocity, $\ddot{{\mathbf {u}}}(t,{\mathbf {X}})$ is the nodal acceleration, $\mathbf {F}({\mathbf {u}},{\mathbf {X}})$ is the deformation gradient, $\mathbf {S}({\mathbf {u}}, {\mathbf {X}})$ is the second Piola–Kirchhoff stress tensor, ${\mathbf {b}}_0({\mathbf {X}})$ represents the body forces and $(\nabla _{{\mathbf {X}}}\;\cdot )$ denotes the divergence operator in the reference configuration. We consider as initial conditions

$$\begin{aligned} {\mathbf {u}}(0,{\mathbf {X}}) = {\mathbf {0}}, \quad \dot{{\mathbf {u}}}(0,{\mathbf {X}}) = {\mathbf {0}}, \end{aligned}$$

and we set ${\mathbf {b}}_0({\mathbf {X}}) = {\mathbf {0}}$ for the sake of simplicity.

We consider the following decomposition of the total stress $\mathbf {S}$:

4 $$\begin{aligned} \mathbf {S}= \mathbf {S}_\mathrm {pas}+ \mathbf {S}_\mathrm {act}, \end{aligned}$$

where $\mathbf {S}_\mathrm {pas}$ and $\mathbf {S}_\mathrm {act}$ denote the passive and active stresses, respectively. Passive stresses are modelled according to the constitutive law

5 $$\begin{aligned} \mathbf {S}_\mathrm {pas}=2\frac{\partial \Psi (\mathbf {C})}{\partial \mathbf {C}} \end{aligned}$$

with $\Psi$ an invariant-based strain-energy function used to model the anisotropic behaviour of cardiac tissue. Numerous constitutive models have been developed in the literature, ranging from simpler transversely-isotropic models to more complex orthotropic laws. In this article, we consider a hyper-elastic and transversely isotropic strain-energy function $\Psi$

6 $$\begin{aligned} \Psi _{\mathrm {Guc}}(\mathbf {C}) = \frac{\kappa }{2} {\left( \log \,J \right) }^2 + \frac{C_\mathrm {Guc}}{2}\left[ \exp (\mathcal {Q})-1\right] \end{aligned}$$

proposed by Guccione et al. [23]. Here, the term in the exponent is

7 $$\begin{aligned} \begin{aligned} \mathcal {Q} =&\, b_{\mathrm {f}} {({\mathbf {f}}_0\cdot \overline{\mathbf {E}}\,{\mathbf {f}}_0)}^2 \\&\,+ b_{\mathrm {t}} \left[ {({\mathbf {s}}_0\cdot \overline{\mathbf {E}}\,{\mathbf {s}}_0)}^2+ {({\mathbf {n}}_0\cdot \overline{\mathbf {E}}\,{\mathbf {n}}_0)}^2+ 2{({\mathbf {s}}_0\cdot \overline{\mathbf {E}}\,{\mathbf {n}}_0)}^2\right] \\&\,+ 2b_{\mathrm {fs}} \left[ {({\mathbf {f}}_0\cdot \overline{\mathbf {E}}\,{\mathbf {s}}_0)}^2+ {({\mathbf {f}}_0\cdot \overline{\mathbf {E}}\,{\mathbf {n}}_0)}^2\right] \end{aligned} \end{aligned}$$

and $\overline{\mathbf {E}}=\frac{1}{2}(\overline{\mathbf{C}}-\mathbf {I})$ is the modified isochoric Green–Lagrange strain tensor, where $\overline{\mathbf{C}} := J^{-2/3} \mathbf {C}$ with $J=\det \mathbf {F}$. Default values of $b_{\mathrm {f}}=18.48$, $b_\mathrm {t}=3.58$, and $b_\mathrm {fs}=1.627$ are used. The parameter $C_\mathrm {Guc}$ is used to fit the LV model to an empirical Klotz relation [24] by a combined unloading and re-inflation procedure [25]. The bulk modulus $\kappa$, which serves as a penalty parameter to enforce nearly incompressible material behaviour, is set as $\kappa = 650\,\hbox {kPa}$.

#### Active cardiac mechanics

Following the approach proposed by [26, 27], we assume that stresses due to active contraction are orthotropic, with full contractile force along the myocyte fibre orientation ${\mathbf {f}}_0$ and 40% contractile force along the sheet orientation ${\mathbf {s}}_0$. In more detail, we define the active stress tensor ${\mathbf {S}}_{\mathrm {a}}$ as

8 $$\begin{aligned} \begin{aligned} {\mathbf {S}}_{\mathrm {a}} =&\, S_{\mathrm {a}} {\left( {\mathbf {f}}_0\cdot {\mathbf {C}}{\mathbf {f}}_0\right) }^{-1} {\mathbf {f}}_0 \otimes {\mathbf {f}}_0\\&+0.4\,S_{\mathrm {a}} {\left( {\mathbf {s}}_0\cdot {\mathbf {C}}{\mathbf {s}}_0\right) }^{-1} {\mathbf {s}}_0 \otimes {\mathbf {s}}_0, \end{aligned} \end{aligned}$$

with $S_\mathrm {a}$ the scalar active stress that describes the contractile force. Active stress generation is modelled using a simplified phenomenological contractile model [28]. Owing to its small number of parameters and its direct relation to clinically measurable quantities such as peak pressure and the maximum rate of rise of pressure, this model is relatively easy to fit [29] and thus suitable for being used in clinical EM modelling studies. In particular, the active stress transient is defined as

9 $$\begin{aligned} \begin{aligned} S_\mathrm {a}(t,\lambda ) =&\, S_\mathrm {peak} \phi (\lambda ) \, \tanh ^2 \left( \frac{t_{s}}{\tau _\mathrm {c}} \right) \tanh ^2 \left( \frac{t_\mathrm {dur} - t_{\mathrm {s}}}{\tau _\mathrm {r}} \right) , \\ {}&\text {for } 0< t_{\mathrm {s}} < t_\mathrm {dur}, \end{aligned} \end{aligned}$$

where

10 $$\begin{aligned} \begin{aligned} \phi =&\tanh (\mathrm {ld} (\lambda - \lambda _0)),\\ \tau _\mathrm {c} =&\, \tau _\mathrm {c_0} + \mathrm {ld}_\mathrm {up}(1-\phi ),\\ t_{\mathrm {s}} =&\, t - t_\mathrm {a} - t_\mathrm {emd} \end{aligned} \end{aligned}$$

and $t_{\mathrm {s}}$ is the onset of contraction, $\phi (\lambda )$ is a nonlinear length-dependent function in which $\lambda$ is the fibre stretch and $\lambda _0$ is the lower limit of fibre stretch below which no further active tension is generated, $t_{\mathrm {a}}$ is the local activation time from Eq. (1), $t_{\mathrm {emd}}$ is the EM delay between the onsets of electrical depolarisation and active stress generation, $S_{\mathrm {peak}}$ is the peak isometric tension, $t_\mathrm {dur}$ is the duration of active stress transient, $\tau _{\mathrm {c}}$ is time constant of contraction, $\tau _{\mathrm {c_0}}$ is the baseline time constant of contraction, $\mathrm {ld}_{\mathrm {up}}$ is the length-dependence of $\tau _{\mathrm {c}}$, $\tau _{\mathrm {r}}$ is the time constant of relaxation, and $\mathrm {ld}$ is the degree of length dependence. Thus, active stresses are only length-dependent in this simplified model, but dependence on fibre velocity $\dot{\lambda }$ is neglected.

### Vascular system

The vascular system imposes a load on the heart and, therefore, affects its mechanical activity. However, the interaction between the heart and the vascular system is bidirectional, i.e., the outflow of blood from a heart cavity and pressure in the cavity depend on the current state of the vascular system, and pressure and flow in the vascular system are determined by the current state of the cavity itself.

The full physics of this interaction is most accurately posed as a fluid-structure interaction (FSI) problem where pressure, $p({\mathbf {X}})$, and flow velocity, ${\mathbf {v}}({\mathbf {X}})$, of the fluid are the coupling variables [30–32]. Any perturbation in blood flow velocity and pressure changes the state of deformation of the heart and attached vessels. As a result, this change in strain implies a change in stress within the myocardial muscle. Conversely, any change in strain or compliance of the attached vessels or of the heart changes the pressure and flow of the fluid.

Even though such a distributed PDE-based approach may accurately describe this interaction, at the level of the whole vascular system it is hardly feasible, for computational and structural reasons. In fact, currently multi-beat simulations using a 3D FSI model solution are not tractable in a clinical time frame. Moreover, the use of 3D models of haemodynamics would require the identification of a much larger number of parameters, that is not easily attainable within clinical constraints.

A reduced-order approximation of the vascular system is used instead in this work, which is based on a 1D model for the vascular system and is particularly suitable for simulating blood flow in the aorta and the larger systemic arteries. The parameters in the vascular model are identified and constrained by imaging-based measurements of the heart and blood flow and invasive blood pressure measurements. In this framework the nonlinear PDE EM model of the heart is coupled to the 1D model of the systemic arteries using the lumped hydrostatic pressure $p_\mathrm {cav}$ in the cardiac cavity and the flow $q_\mathrm {cav}$ out of the cavity into the vascular system as coupling variables.

#### 1D mathematical model of the blood flow circulation in the arterial system

**Fig. 1:**
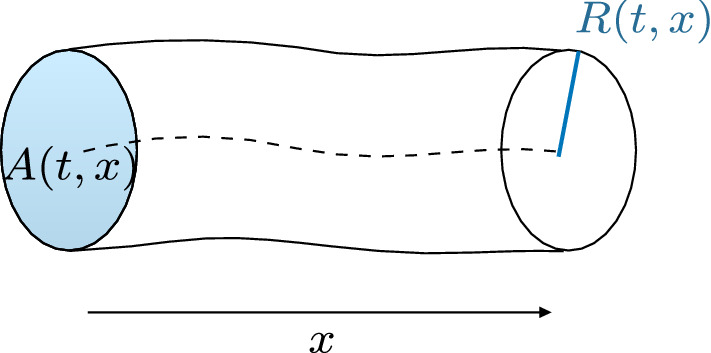
Description of a 1D compliant arterial segment with properties described by a single axial coordinate *x*

The 1D equations of arterial blood flow can be derived from the Navier-Stokes equations after assuming axisymmetric flow in a cylindrical tube with thin wall [9], see Fig. 1, obtaining

11 $$\begin{aligned} {\left\{ \begin{array}{ll} \displaystyle \frac{\partial A}{\partial t}+\frac{\partial Q}{\partial x}=0 \\[6pt] \displaystyle \frac{\partial Q}{\partial t} + \frac{\partial }{\partial x}\Big (\alpha \frac{Q^2}{A} \Big )+\frac{A}{\rho } \frac{\partial P}{\partial x}= \frac{f}{\rho }, \end{array}\right. } \end{aligned}$$

where *A*(*x*, *t*) is the *cross-sectional area* of the lumen, *Q*(*x*, *t*) is the *average flux* and *P*(*x*, *t*) is the *average internal pressure* over the cross-section. Note that in the momentum balance a momentum flux correction factor $\alpha$ (also known as Coriolis coefficient) is introduced, defined as

$$\begin{aligned} \alpha = \frac{1}{A\, \bar{u}^2}\int _{S(x,t)} u_x^2(x,t) \, \mathrm {d}\sigma . \end{aligned}$$

An energy inequality that bounds a measure of the energy of the hyperbolic system was derived in [33]. Alternatively, it is possible to rewrite the system in terms of the variables (*A*, *u*). In more detail, if we assume a flat velocity profile in the convective acceleration term, i.e. $\alpha = 1$, we obtain the system

12 $$\begin{aligned} {\left\{ \begin{array}{ll} \displaystyle \frac{\partial A}{\partial t}+\frac{\partial (Au)}{\partial x}=0 \\[6pt] \displaystyle \frac{\partial u}{\partial t} + u\frac{\partial u}{\partial x}+\frac{1}{\rho } \frac{\partial P}{\partial x}= \frac{f}{\rho A}, \end{array}\right. } \end{aligned}$$

where *A*(*x*, *t*) is the *cross-sectional area* of the lumen, *u*(*x*, *t*) is the *average axial velocity* and *P*(*x*, *t*) is the *average internal pressure* over the cross-section.

For a constant velocity profile satisfying the no-slip condition, the friction force per unit length is $f(x,t)= -\eta \,u(x,t)$, where $\eta$ is a coefficient depending on the blood viscosity and the *average axial velocity*. As System (12) is composed by two equations and three unknowns (*A*, *u*, *P*), a closure condition is needed, namely the so-called *tube law*. In this work, we only take into account the arterial circulation and we consider a Voigt-type visco-elastic constitutive law for the arterial wall [20]:

13 $$\begin{aligned} \begin{aligned} P(x,t)=&\, P_{ext}(x,t)+K\,\phi \bigl (A(x,t),A_0(x)\bigr )\\&\ + G \,\Psi \bigl (A(x,t)\bigr ), \end{aligned} \end{aligned}$$

where *K* and *G* are parameters depending on the *wall stiffness* and the wall *viscosity*, respectively.

The purely elastic contribution reads

$$\begin{aligned} \phi = \frac{\sqrt{A}- \sqrt{A_0}}{A_0}, \ K = \frac{4}{3}\sqrt{\pi }Eh,\ \text {and}\ \Psi =0, \end{aligned}$$

with *E* the Young modulus and *h* the wall thickness of the vessel. Voigt-type visco-elastic effects are considered by setting

$$\begin{aligned} \Psi = \frac{1}{A_0\sqrt{A}} \frac{\partial A}{\partial t}, \quad G = \frac{2}{3} \sqrt{\pi }\varphi h, \end{aligned}$$

with $\varphi (x)$ the wall viscosity.

### 3D-1D coupling

Various approaches can be envisaged to perform the coupling between a (3D) cardiac model and a vascular model. In practice, the problem consists in finding the new state of deformation ${\mathbf {u}}^{n+1}$ as a function of the pressure $p^{n+1}$ in the cavity applied as a Neumann boundary condition at the cavitary surface. This unknown pressure needs to be appropriately determined. Two configurations need to be captured by the model:

- When all valves are closed, the cavity is in an isovolumetric state, i.e., the muscle enclosing the cavity may deform, but the volume has to remain constant. Consequently, during an isovolumetric phase the pressure $p^{n+1}$ in the cavity needs to adjust to the variation over time of active stresses, in order to maintain the cavitary volume constant.
- When one valve is open or regurging, there is a change in cavitary volume. In this configuration the pressure $p^{n+1}$ is regulated by the state of the vascular system or of a connected cavity. Therefore, $p^{n+1}$ needs to be estimated by matching mechanical deformation and state of the system. In fact, pressure $p^{n+1}$ in the cardiovascular system depends on flow, which depends in turn by cardiac deformation. As a consequence, the heart and vascular models are tightly bidirectionally coupled.

From a mathematical perspective, this coupling can be performed in two ways. The simplest approach is to prescribe $p^{n+1}$ explicitly. Then, the coupling is performed during the ejection phase by updating flow and flow rate based on the current prediction on the change in the state of deformation associated with the currently predicted pressure $p^{n+1}$. In particular, the pressure boundary condition in each nonlinear solver step is modified within each iteration ${\nu }$. Note that the new prediction $p^{n+1}_{\nu +1}$ is then explicitly prescribed as a Neumann boundary condition. Therefore, this explicit and partitioned approach may introduce some inaccuracies during ejection phases and might lead to instabilities during isovolumetric phases [17]. Such instabilities arise from the difficulty of estimating the change in pressure necessary to keep the volume constant. In fact, a knowledge on cavitary elastance is required to know the $p-V$ relation of the cavity at this given point in time, that is not available. Therefore, iterative estimates are needed to gradually inflate or deflate a cavity to its prescribed volume. However, since the elastance properties of the cavities are highly nonlinear, an overestimation may induce oscillations, whereas an underestimation may induce very slow convergence and prohibitively large numbers of Newton iterations. A more sophisticated approach consists in treating $p^{n+1}$ as an unknown. Thus, an additional equation is required to close the system, leading to a saddle point formulation and a block system to be solved (monolithic approach).

Following the approach for 3D-0D coupling proposed in [34] and inspired by [14, 35], the coupling condition between an EM model of the heart cavities and a reduced-order vascular model that we consider in this work imposes that the volume change in each cavity (left ventricle LV, right ventricle RV, left atrium LA and right atrium RA) is balanced with the volume change in the attached vascular system. For the sake of generality, we cast in what follows the general framework to couple a four-chamber heart model with a closed-loop vascular system. Let us consider that approximately, at a given time-point, we have constant pressures in each cavity $p_c$, with $c\in \{\mathrm {LV,RV,LA,RA}\}$. Then, the following equations are the starting point of the method:

14 $$\begin{aligned} V_c^\mathrm {heart}({\mathbf {u}})-V_c^\mathrm {CS}(p_c)=0, \end{aligned}$$

where $V_c^\mathrm {heart}({\mathbf {u}})$ is the cavity volume computed with the deformation using Eq. (15), and $V_c^\mathrm {CS}(p_c)$ is the volume as predicted by the blood flow model for the intra-cavitary pressure $p_c$. We write $\underline{p}_{c} ={[p_{c}]}_{c\in \{\mathrm {LV,RV,LA,RA}\}}$ for the vector of up to $1 \le N_\mathrm {cav} \le 4$ pressure unknowns.

Note, however, that in a purely EM simulation framework the fluid domain is not explicitly modelled. Therefore, the cavitary volume $V_c^\mathrm {heart}({\mathbf {u}})$ is not discretised. Instead, only the surface enclosing the cavitary volume is known. Nonetheless, if we assume that the entire surface of the cavitary volume is available, including the faces representing the valves, then we can compute for each instant *t* of the cardiac cycle the enclosed volume $V^\mathrm {heart}_c({\mathbf {u}})$ from the surface $\Gamma _c$ by means of the divergence theorem

15 $$\begin{aligned} V^\mathrm {heart}_c({\mathbf {u}})=\frac{1}{3}\int _{\Gamma _c} {\mathbf {u}}(t)\cdot {\mathbf {n}}\,\mathrm {d}\Gamma _c. \end{aligned}$$

Then, the approach used to evolve the full system of equations of nonlinear elasticity Eq.(14) together with the coupling condition Eq.(15) is based on the resolution of a saddle-point problem in the variables $({\mathbf {u}}, \underline{p} )$, corresponding to the displacement and the pressure in the cavity:

16 $$\begin{aligned} \begin{aligned} \langle \mathcal {A}_0({\mathbf {u}}),{\mathbf {v}}\rangle _{\Omega _0} - \langle \mathcal {F}_0({\mathbf {u}},p_c),{\mathbf {v}}\rangle _{\Omega _0}&={\mathbf {0}}\\ \langle V_c^\mathrm {heart}({\mathbf {u}}),q\rangle _{\Omega _0} - \langle V_c^\mathrm {CS}(p_c), q\rangle _{\Omega _0}&= 0, \end{aligned} \end{aligned}$$

which is valid for all vector fields ${\mathbf {v}}$ smooth enough and satisfying the given boundary conditions, test functions *q* that are 1 for the cavity *c* and 0 otherwise, the duality pairing $\langle \cdot ,\cdot \rangle _{\Omega _0}$, and cavities $c\in \{\mathrm {LV,RV,LA,RA}\}$. Note that $\langle A_0({\mathbf {u}}), {\mathbf {v}}\rangle _{\Omega _0}$ can be physically interpreted as the rate of internal mechanical work, whereas $\langle {\mathbf {\mathcal {F}}}_0({\mathbf {u}}, p_c), {\mathbf {v}}\rangle _{\Omega _0}$ takes into account the contribution of pressure loads. Using a Galerkin discretisation and the Newton method, the problem to be solved at each Newton–Raphson step becomes a block system in order to find $\delta \underline{u}\in \mathbb {R}^{3N}$ and $\delta \underline{p} \in \mathbb {R}^{N_\mathrm {cav}}$ such that:

17 $$\begin{aligned} \begin{aligned} \begin{pmatrix} ({\mathbf {A}}'-{\mathbf {M}}')(\underline{u}^k, \underline{p}_c^k) &{} {\mathbf {B}}'_\mathrm {p}(\underline{u}^k) \\ {\mathbf {B}}'_{{\mathbf {u}}}(\underline{u}^k) &{} {\mathbf {C}}'(\underline{p}_c^k) \\ \end{pmatrix} \begin{pmatrix} \delta \underline{u}\\ \delta \underline{p}_c \end{pmatrix} \\ = - \begin{pmatrix} \underline{A}(\underline{u}^k)-\underline{B}_\mathrm {p}(\underline{u}^k,\underline{p}_c^k) \\ \underline{V}_c^\mathrm {heart}(\underline{u}^k) -\underline{V}_c^\mathrm {CS}(\underline{p}_c^k) \end{pmatrix},\\ \end{aligned} \end{aligned}$$

with the updates

18 $$\begin{aligned} \underline{u}^{k+1} = \underline{u}^{k} + \delta \underline{u},\quad \underline{p}_c^{k+1} = \underline{p}_c^k + \delta \underline{p}_c, \end{aligned}$$

and the solution vectors $\underline{u}^k\in \mathbb {R}^{3N}$ and $\underline{p}_c^k\in \mathbb {R}^{N_\mathrm {cav}}$ at the *k*-th Newton step. In particular, we can retrieve the expression of ${\mathbf {C}}'(\underline{p}_c^k)$ and $\underline{V}^\mathrm {CS}(\underline{p}_c^k)$ from the equations of the vascular model, whereas the term ${\mathbf {B}}'_\mathrm {u}(\underline{u}^k)$ only depends on the contribution of the EM model of the heart. In more detail, ${\mathbf {C}}'(\underline{p}_c^k)$ is approximated by a discrete derivative (finite difference) of the volume ${\text {V}}^\mathrm {CS}$ with respect to the cavity pressure. We refer the reader to Appendix 1 for further information on the coupling strategy and finite element formulation, respectively.

**Fig. 2:**
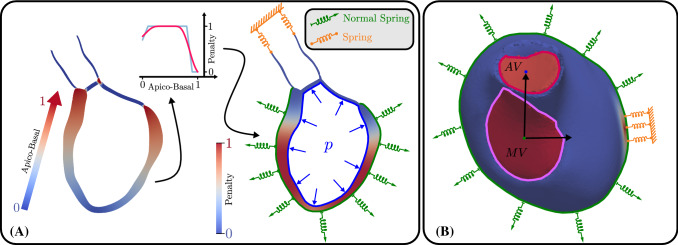
Boundary conditions applied to the LV models. With permission from  [38]

Isovolumetric Phases During the isovolumetric phases we can state that

$$\begin{aligned} {\text {V}}^\mathrm {heart}_c({\mathbf {u}})-V_0=0, \end{aligned}$$

i.e., the volume of each cavity ${V}^\mathrm {heart}_c({\mathbf {u}})$ equals an initial volume $V_0$ and does not change during the isovolumetric phase. Hence, in system (17) the matrix ${\mathbf {C}}'(\underline{p}^k_c)={\mathbf {0}}$ and $V^{\mathrm {CS}}(\underline{p}^k_c)=V_{0}$.

### Boundary Conditions

Mechanics boundary conditions at the LV The boundary of the left ventricular model is decomposed in three components, i.e., $\partial \Omega _0=\overline{\Gamma }_{\mathrm {endo},0} \cup \overline{\Gamma }_{\mathrm {epi},0}\cup \overline{\Gamma }_{\mathrm {base},0}$, where $\overline{\Gamma }_{\mathrm {endo},0}$ denotes the endocardium, $\overline{\Gamma }_{\mathrm {epi},0}$ the epicardium, and $\overline{\Gamma }_{\mathrm {base},0}$ the base of the ventricle. At the endocardium normal stress boundary conditions are imposed:

19 $$\begin{aligned} \begin{aligned} {\mathbf {F}}{\mathbf {S}}({\mathbf {u}},{\mathbf {X}})\,{{\mathbf {n}}^{\mathrm {out}}_0}({\mathbf {X}}) = -p(t) J{\mathbf {F}}^{-\top }{{\mathbf {n}}^{\mathrm {out}}_0}({\mathbf {X}})\\ \text {on}\quad {\Gamma }_{\mathrm {endo},0}\times (0,T) \end{aligned} \end{aligned}$$

with ${{\mathbf {n}}^{\mathrm {out}}_0}$ the outer normal vector. Omni-directional spring type boundary conditions constrain the ventricle at the basal cut plane $\overline{\Gamma }_{\mathrm {base},0}$ [36], and spatially varying normal Robin boundary conditions are applied at the epicardium $\overline{\Gamma }_{\mathrm {epi},0}$ [37] to simulate the pericardial mechanical constrains. The different BCs applied to the LV models are summarised in Fig. 2. The springs attached to the aortic rim and at the pericardium are shown in Fig. 2A, as well as the pressure BC in the cavity at the endocardial surface. In more detail, the pericardial springs penalise displacement only in normal direction and are gradually scaled from the apex to the base. Therefore, the distance in apico-basal direction is used to create a penalty map, see Fig. 2A. To avoid non-physiological rotation, further springs are attached to the septum, see Fig. 2B. The location of the septal springs is selected automatically by constructing a local coordinate system spanned by the centres of the apical region, the mitral and the aortic valve.

Mechanics boundary conditions of the arterial system The arterial network is connected to the LV cavity through a model of aortic valve dynamics, based on Bernoulli’s equation [39]. See Appendix 1 for further details on the valve dynamics model.

Arterial 1D models must be truncated after a certain number of generations of bifurcations, since the relative size of red blood cells to vessel diameter increases, therefore the assumptions made in 1D modelling that blood is a continuum and a Newtonian fluid fail. Moreover, contrary to large arteries, fluid resistance dominates over wall compliance and fluid inertia in smaller vessels. For these reasons, linear lumped parameter models are commonly employed to simulate the effect of peripheral vessels on pulse wave propagation in larger arteries [20, 40]. Such models are obtained by averaging Eq. (12) over the length of a vessel and considering some simplifying hypotheses, such as neglecting the convective term in the momentum equation. A suitable lumped parameter model for our purposes is composed of one capacitor and two resistors [41]. In more detail, the first resistance *Z* corresponds to the characteristic aortic impedance. This is connected in series with a parallel combination of the peripheral arterial resistance *R* and compliance *C*. We define $$P_{out} $$ as the pressure at which flow in the microcirculation is equal to zero. The resulting model is governed by

20 $$\begin{aligned} Q \Big (1 + \frac{Z}{R}\Big ) + C\, Z \frac{\partial {Q}}{\partial {t}} = \frac{P - P_{out}}{R} + C \frac{\partial {P_e}}{\partial {t}}. \end{aligned}$$

### Numerical framework

Spatio-temporal discretisation of all PDEs and the solvers for the resulting systems of equations of the cardiac EM model are based on the Cardiac Arrhythmia Research Package (CARPentry)  [1, 21, 42], built upon extensions of the freely available openCARP electrophysiology framework (http://www.opencarp.org). For numerical details on the finite element discretisation, we refer the reader to Appendix 1.

Concerning the one-dimensional model of the arterial system, in this study we used the solver Nektar1D (http://haemod.uk/nektar) [20], which is based on a FEM discretisation (discontinuous Galerkin) in space and finite difference (explicit second-order Adams–Bashforth) in time of Equations (12) and (13).

The coupling scheme and a C++ arterial system module have been embedded in CARPentry, based on the software Nektar1D, in order to preserve computational efficiency and strong scalability. A Schur complement approach (see Appendix B.2) is considered to cast the coupling problem in a pure displacement formulation, in order to use solver methods already implemented [1]. A generalised minimal residual method (GMRES) with an relative error reduction of $\epsilon =10^{-8}$ is employed. The library *PETSc* [43] and the incorporated solver suite *hypre/BoomerAMG* [44] are used for efficient preconditioning and solving of the linear systems. A generalised-$\alpha$ scheme is considered for time integration, with spectral radius $\rho _\infty =0$ and damping parameters $\beta _\mathrm {mass}={0.1}$, $\beta _\mathrm {stiff}={0.1}$, see [34] for further information.

### Model parameterisation

In order to guarantee the stability of the coupled system, the solution of the arterial 1D model is initialised until a periodic solution is reached. This is achieved by performing 20 cycles of the arterial model alone prior to coupling with the heart 3D model, with a flow or pressure profile provided as an inlet boundary condition. For example, a flow or pressure waveform clinically measured at the aortic root can be used as a boundary condition, if available. Otherwise, analytical functions can be prescribed [45]. For the current study, we considered two different choices: an invasively-recorded pressure measurement at the aorta and an analytical function of the flow waveform calibrated to match measured peak flow, opening, maximum and closing pressure at the aorta.

**Fig. 3:**
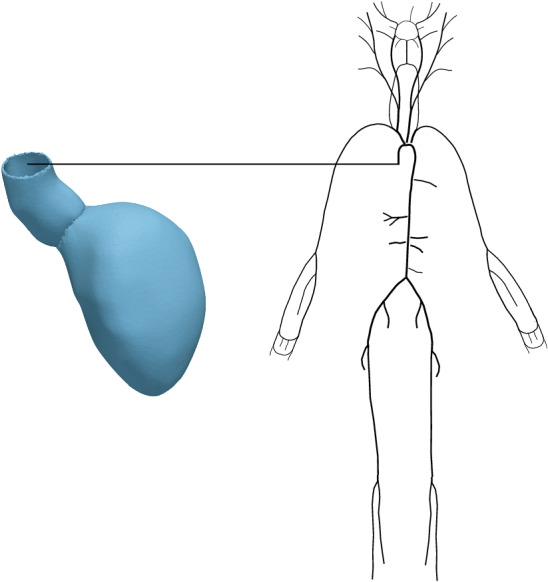
Left: Computational mesh of LV derived from patient-specific image-based clinical data. Right: 1D arterial network

The coupled system is solved using a Newton method with a maximal number of steps $k_{\max }=10$ and an absolute $\ell _2$ norm error reduction of the residual of $\epsilon =10^{-6}$. To illustrate the performance of the coupling method we consider a system consisting of a 3D EM model of the LV and for the 1D arterial model we analyse different geometries, either consisting of a single arterial segment or composed by more complex networks including the larger systemic arteries. To reproduce a realistic case, the LV computational domain is adapted from a patient-specific 3D-whole-heart-MRI scan collected and post-processed in a previous study, see Figure 3.

The biomechanical parameters of the solid and fluid models under baseline conditions are calibrated to match a patient-specific set of *in vivo* measurements at specific instants of the cardiac cycle for the same subject, see [38] for further information.

The same pressure-volume data is used for the initialisation of the 3D EM model. All parameters used for the baseline case are summarised in Table 1 and  Table 2.

In the first test cases proposed in this work we consider an arterial network consisting of a segment of the human upper thoracic aorta, equipped with a 3-element Windkessel model as a terminal boundary condition. A more complex arterial network [46] is also considered in Sect. 3.2.3.

**Table 1:** Input parameters for the 3D PDE model of the left ventricle. Adjusted to match patient-specific data

Parameter	Value	Unit	Description
*Passive biomechanics*			
$\rho _0$	1060.0	$\hbox {kg}/\hbox {m}^{3}$	Tissue density
$\kappa$	650	kPa	Bulk modulus
*a*	0.8	kPa	Stiffness scaling
$b_\mathrm {ff}$	5.0	[-]	Fibre strain scaling
$b_\mathrm {ss}$	6.0	[-]	Cross-fibre in-plain strain scaling
$b_\mathrm {nn}$	3.0	[-]	Radial strain scaling
$b_\mathrm {fs}$	10.0	[-]	Shear strain in fibre-sheet plane scaling
$b_\mathrm {fn}$	2.0	[-]	Shear strain in fibre-radial plane scaling
$b_\mathrm {ns}$	2.0	[-]	Shear strain in transverse plane scaling
*Active biomechanics*			
$\lambda _0$	0.7	ms	Onset of contraction
$V_\mathrm {m,Thresh}$	$-$60.0	mV	Membrane potential threshold
$t_\mathrm {emd}$	15.0	ms	EM delay
$S_\mathrm {peak}$	60	kPa	Peak isometric tension
$t_\mathrm {dur}$	575.0	ms	Duration of active contraction
$\tau _{c_0}$	105.0	ms	Baseline time constant of contraction
$\tau _\mathrm {r}$	90.0	ms	Time constant of relaxation
$\mathrm {ld}$	35.0	[-]	Degree of length-dependence
$\mathrm {ld}_\mathrm {up}$	100.0	ms	Length-dependence of upstroke time
*Electrophysiology*			
$t_\mathrm {cycle}$	1.231	s	Cycle time ($=1/\mathrm {heartrate}$)
AA delay	20.0	ms	Inter-atrial conduction delay
AV delay	100.0	ms	Atrioventricular conduction delay
VV delay	0.0	ms	Inter-ventricular conduction delay
$(v_\mathrm {f},v_\mathrm {s},v_\mathrm {n})$	(0.6, 0.4, 0.2)	m/s	Conduction velocities
$(g_\mathrm {f},g_\mathrm {s},g_\mathrm {n})$	(0.44, 0.54, 0.54)	m/s	Conductivities in LV
$\beta$	1/1400	$\hbox {cm}^{-1}$	Membrane surface-to-volume ratio
$C_\mathrm {m}$	1	$\upmu \hbox {F}/\hbox {cm}^{2}$	Membrane capacitance

**Table 2:** Input parameters for the 1D PDE model of the arterial circulation

Parameter	Value	Unit	Description
$\rho _0$	1060.0	$\hbox {kg}/\hbox {m}^{3}$	Blood density
$\nu$	4e-3	Pa s	Blood viscosity
$\alpha$	1.1	[-]	Coriolis coefficient (momentum equation)
*G*	1	kPa	Vessel wall viscosity

## Results

### Stability assessment of the coupling method

To analyse the stability of the proposed approach, we compared the results of simulations with a varying time resolution for the 1D blood flow scheme or the 3D LV model, respectively. Taking into account the intrinsic time-step limitation necessary to ensure stability of the 1D blood flow scheme, associated with a CFL condition [47], we did not consider a time step $\mathrm {dt1D}$ higher than $1 \times 10^{-3}\hbox {s}$. In more detail, in the first test case we considered $\mathrm {dt1D}\in \{5 \times 10^{-4}\hbox {s}, 1 \times 10^{-4}\hbox {s}, 5 \times 10^{-5}\hbox {s}\}$, while keeping the time resolution for the 3D LV model constant at $\mathrm {dt3D}=1 \times 10^{-3}\hbox {s}$. The arterial system is represented in this case by one vessel segment of $200\,\hbox {mm}$ and an RCR Windkessel model for terminal boundary conditions. We emphasise that the coupling is always performed at the time resolution of the 3D EM model.

**Fig. 4:**
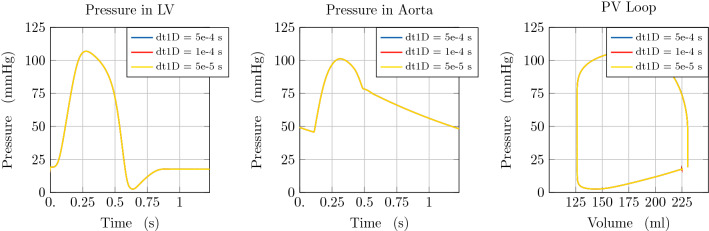
Illustration of model predictions. Comparison of model predictions considering different time resolutions (dt1D) for the 1D arterial model. Left: Pressure with time in the LV. Middle: Pressure with time at the inlet of the aorta. Right: Pressure-volume loop in the LV

Figure 4 demonstrates that solutions remain unchanged when considering a different time discretisation of the arterial model. Therefore, we consider the lowest temporal resolution ensuring stability of the 1D numerical scheme.

**Fig. 5:**
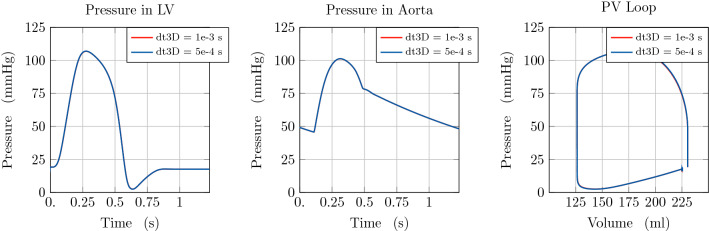
Illustration of model predictions. Comparison of model predictions considering different time resolutions (dt3D) for the 3D cardiac model. Left: Pressure with time in the LV. Middle: Pressure with time at the inlet of the aorta. Right: Pressure-volume loop in the LV

As a second example, we performed two simulations considering a varying time resolution for the 3D heart model, $\mathrm {dt3D} \in \{1 \times 10^{-3}\hbox {s}, 5 \times 10^{-4}\hbox {s}\}$, keeping the same temporal resolution $\mathrm {dt1D} = 1 \times 10^{-4}\hbox {s}$ for the 1D arterial model. Figure 5 shows that, also in this case, solutions remain unchanged when considering a different time discretisation of the 3D heart model. Thus, we consider $\mathrm {dt3D} = 1 \times 10^{-3}\hbox {s}$ in what follows. We refer to [1] for a more detailed convergence testing of the cardiac model alone.

### Prediction of pulse wave propagation effects on cardiac function

To test the ability of the coupled model to predict the effect of pulse wave propagation on cardiac EM we considered three test cases, ranging from an idealised aortic segment with a constant radius, to a pathological condition called aortic coarctation and a network composed of 116 arterial segments.

#### Idealised aortic segment with constant radius

The first example illustrates the effect of arterial stiffening on haemodynamics and LV function. In particular, we considered a 3D LV solid model coupled to a vessel segment with constant radius and a length of 126 mm with lumped terminal boundary conditions. For the baseline case we set the vessel wall stiffness described by the Young modulus to $E =0.25 \times 10^{6}\hbox {Pa}$. For two test cases with increased stiffness we set the Young modulus *E* to $0.50\times 10^{6}\hbox {Pa}$ and $0.75\times 10^{6}\hbox {Pa}$, respectively. The 1D blood flow model was initialised using an inflow boundary condition. For the simulations in this test case we fixed $\mathrm {dt1D} =1 \times 10^{-4}\hbox {s}$.

**Fig. 6:**
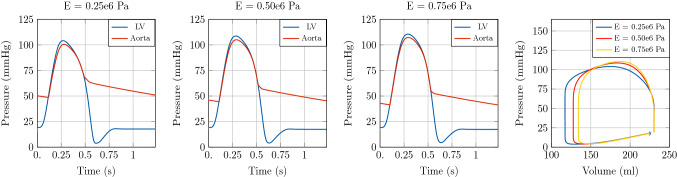
Illustration of model predictions. Test case 1: Idealised aortic segment with constant radius. Left: Pressure with time in the LV and at the inlet of the aorta. Right: Pressure-volume loop in the LV. Comparison of model predictions considering three different Young’s modulus *E* in the 1D blood flow model

Simulation results of the coupled model are shown in Fig. 6, where the time-varying pressure in the LV and at the inlet of the aortic segment are compared for the different stiffness values. It can be observed that an increase in *E* produced an increase in peak pressure and a substantial change in the shape of the pressure wave. This is due to the fact that the stiffening of the vessel wall affects pulse wave propagation in the vessel itself. In particular, the increase of aortic stiffness is associated with a premature rise and decay of the pressure wave and the well-known phenomenon of peak pressure augmentation [48].

**Fig. 7:**
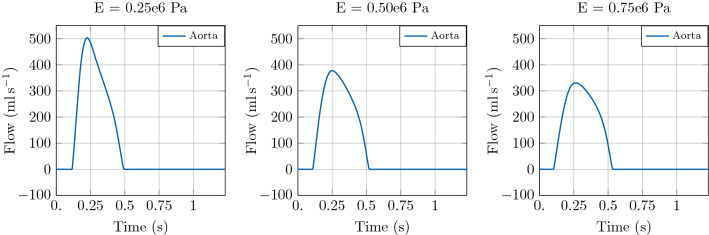
Illustration of model predictions. Test case 1: Idealised aortic segment with constant radius. Flow with time in the LV and at the inlet of the aorta. Comparison of model predictions considering three different Young’s modulus *E* in the 1D blood flow model

The increased stiffness of the aortic vessel has a significant impact on LV function, see the PV loop in Fig. 6 and  Fig. 7. The stiffening of the arterial vessel is associated with an increase in end-systolic volume (ESV) at unchanged end-diastolic volume (EDV), leading to a reduction of stroke volume (SV).

#### Stenotic aortic segment

As a second example, we considered a pathological configuration of aortic coarctation, that is a congenital defect consisting of a local narrowing of the aortic lumen (most commonly in the aortic arch). To this end, we considered an arterial segment of 126 mm endowed with a stenosis model [49], with a 30% stenosis halfway of its length. Terminal boundary conditions are given by an RCR Windkessel model. In order to explore the effect of arterial stiffening in this configuration, we considered a baseline case with $E = 0.25\times 10^{6}\hbox {Pa}$ and two test cases with *E* equal to $0.50\times 10^{6}\hbox {Pa}$ and $0.75\times 10^{6}\hbox {Pa}$, respectively. We set $\mathrm {dt1D} =1\times 10^{-5}\hbox {s}$ for stability considerations.

**Fig. 8:**
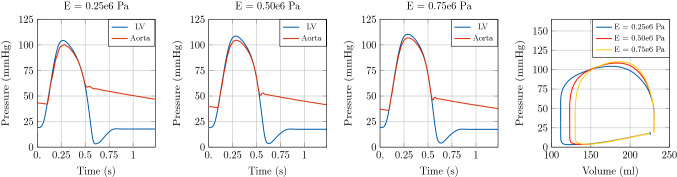
Illustration of model predictions. Test case 2: Aortic segment with coarctation. Left: Pressure with time in the LV and at the inlet of the aorta. Right: Pressure-volume loop in the LV. Comparison of model predictions considering three different Young’s modulus *E* in the 1D blood flow model

**Fig. 9:**
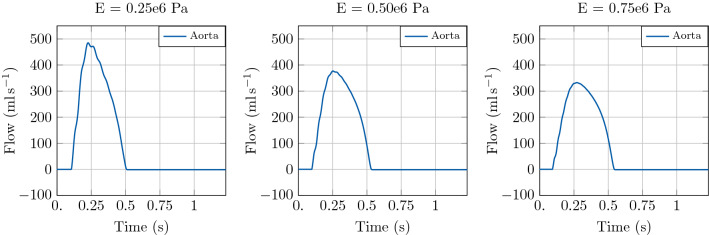
Illustration of model predictions. Test case 2: Aortic segment with coarctation. Flow with time in the LV and at the inlet of the aorta. Comparison of model predictions considering three different Young’s modulus *E* in the 1D blood flow model

Also in this example, an increase in aortic stiffness leads to an increase in peak pressure and a variation in the pressure profile, see Fig. 8. The effect of vessel stiffness on LV function is shown in the PV loop of Fig. 8 and it consists in a reduction in SV caused by an increase of the ESV. The associated flow profile for this test case is depicted in Fig. 9.

**Fig. 10:**
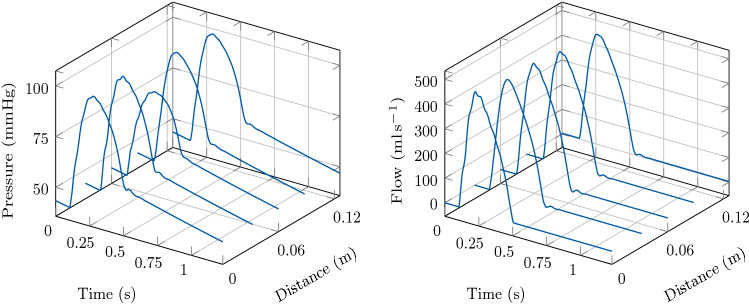
Illustration of model predictions. Test case 2: Aortic segment with coarctation. Pressure and flow with time along the aortic segment. Young’s modulus $E = 0.25\times 10^{6}\hbox {Pa}$

For the sake of completeness we show the variation of pressure and flow signals along the distance of the 1D vessel for the case $E = 0.25\times 10^{6}\hbox {Pa}$. In particular, we show the model predictions of the 3D-1D coupled model in Fig. 10.

**Fig. 11:**
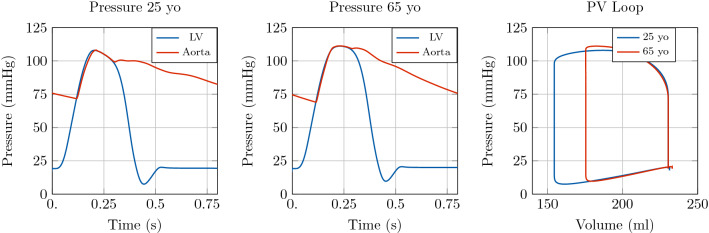
Illustration of model predictions. Test case 3: Network with the largest 116 systemic arteries. Left: Pressure with time in the LV and at the inlet of the aorta. Right: Pressure-volume loop in the LV. Comparison of model predictions considering different vascular properties in the arterial 1D model corresponding to a 25 yo and a 65 yo subject, respectively

#### 116 aortic segments

In order to explore the effect of bifurcations on pulse wave reflections and their impact on LV function the 3D EM model of the LV was coupled to a more complex model of the arterial system. To this end, as a third example we coupled the 3D model to a network consisting of 116 arterial segments. Moreover, we explored two different physiological configurations for the 1D model, corresponding to a 25 year-old subject and a 65 year-old subject. We refer to [46] for further detail on the 1D networks considered. Concerning the 3D heart model, we considered the same parameter values as in the previous test cases, listed in Table 1, with some exceptions. In particular, the heart cycle period was set to $800 \hbox {ms}$ in both configurations, and the parameters of the active stress law were adapted according to the new heart cycle period. We set $\mathrm {dt1D} =1\times 10^{-5}\hbox {s}$ in this test case as well.

**Fig. 12:**
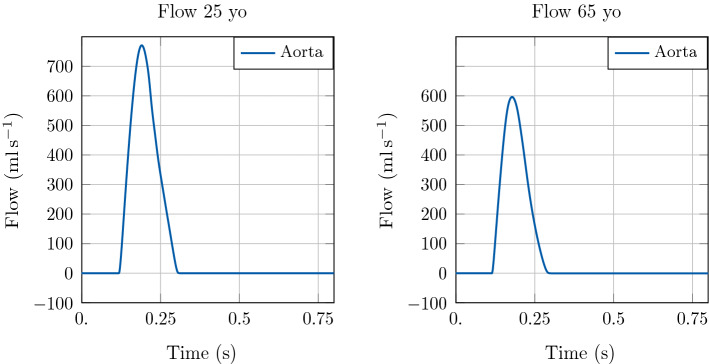
Illustration of model predictions. Test case 3: Network with the largest 116 systemic arteries. Flow with time in the LV and at the inlet of the aorta. Comparison of model predictions considering different vascular properties in the arterial 1D model corresponding to a 25 yo and a 65 yo subject, respectively

Also in this example, the increase in aortic stiffness and systemic vascular resistance associated with healthy ageing is reflected in an augmentation of the peak pressure and a variation in the pressure profile, see Fig. 11. The effect of ageing on LV function is also considerable, as shown in Figs. 11 and 12. In particular, we can see a reduction in SV caused by an increase in ESV.

**Fig. 13:**
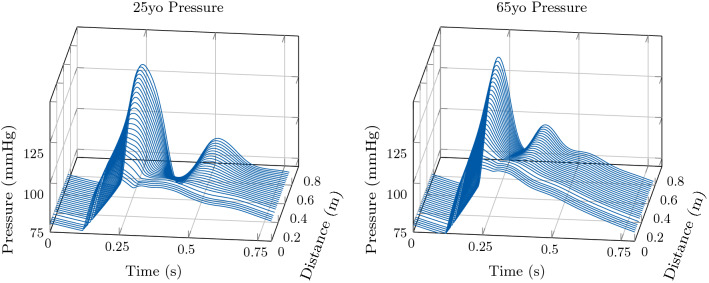
Illustration of model predictions. Test case 3: Network with the largest 116 systemic arteries. Change in the pressure waveform from the aortic root to the right brachial artery (in the arm). Comparison of model predictions considering different vascular properties in the arterial 1D model corresponding to a 25 yo and a 65 yo subject, respectively

For this test case we can show the effect of pulse pressure amplification, i.e. the amplification in pulse pressure signal towards more distal locations, associated with reflections at bifurcation sites. For the sake of illustration, in Fig. 13 we consider the change in the pressure waveform from the aortic root to the right brachial artery. As expected, pulse pressure amplification was more pronounced in the younger subject than in the 65-yo subject.

## Discussion

In this methodological study we have developed a numerical scheme that allows to couple a state-of-the-art 3D EM model of cardiac function to a 1D model of arterial blood flow. For the first time, a personalised coupled 3D-1D model of LV and arterial system is built and used in numerical benchmarks to demonstrate robustness and accuracy of our scheme over a range of time steps. We have validated the coupled model by investigating its physiological response to variations in the arterial system influencing pulse wave propagation, such as aortic stiffening, aortic stenosis or bifurcations inducing wave reflections.

First, we have demonstrated the feasibility of the proposed approach by coupling a patient-specific 3D LV model to a 1D model of the aorta and shown the ability of the model to correctly predict known physiological behaviours associated with arterial wall stiffening both in a healthy case and in the pathological case of aortic coarctation.

Furthermore, we have shown an application to the coupling of the 3D LV model with a complex network composed by 116 arterial segments, in order to study the effect of bifurcations and tapering on pulse wave reflections and their impact on cardiovascular function.

### Prediction of pulse wave propagation effects on cardiac function

The results in Sect. 3 show an augmentation of peak pressure in the LV and pulse pressure in the aorta and a substantial variation in the shape of the pressure trace as a consequence of stiffer vessel walls. Peak pressure in the LV increases with increasing E, since there is a higher afterload. In the aorta, the pulse pressure increases with increasing Young’s modulus E, since the aortic model becomes less compliant.

Physiologically, the aortic pressure waveform is composed of two additive components: first, a forward propagating wave generated by LV contraction and second, a backward propagating wave from the periphery back to the aortic root. From this, the behaviour observed in the simulations is to be expected as the increased aortic stiffness generates a faster pulse wave propagation. Hence, this causes a premature arrival of the forward propagating wave and, in turn, of the backward propagating wave, entailing peak pressure augmentation [48].

In addition, the stiffening of the arterial vessel is related to an increase in ESV. The main reason for this effect is that a lower compliance of the vessel, i.e., its ability to distend and increase volume with increasing transmural pressure, is responsible for a reduction of blood ejected by the heart chamber, thus producing a greater ESV. Since EDV is influenced by the cardiac passive behaviour and preload rather than by afterload, this volume does not vary when the aortic vessel properties change. Hence, the stiffening of the aortic vessel leads to a reduction in SV.

We have shown that the proposed 3D-1D model is capable to reproduce the impact of increased stiffness and pulsatile load in the circulation and their effect on heart performance. These results confirm that such models are particularly suited to explore the effects of changes in pulse wave propagation, which can hardly be captured using lumped parameter models [20]. Also, effects of these interactions are very complex to study *in vivo* due to the need for high fidelity and simultaneous measurements of cardiac and vascular haemodynamics quantities at several locations, together with the capability to assess chamber interactions [50]. Hence, 3D-1D models are of high relevance to investigate interactions between vascular waves and cardiac chamber function.

Overall, a coupling to 1D models should be preferred over a coupling to lumped parameter models for clinical applications that are profoundly influenced by disrupted blood flow propagation, e.g., aortic coarctation pathologies and pulmonary arterial hypertension.

### Numerical aspects

Computationally, the 3D-1D approach is comparable to 3D-0D models, as the extra cost of solving the 1D model is essentially negligible compared to the cost of the 3D model. As such, whenever wave transmission effects are to be investigated, the 3D-1D model is preferable as it can be used instead of a 3D-0D model as in [34] without any computational penalties. Further, we emphasise that the implementation of the coupling framework is not constrained to the specific choice of the 1D solver used for the solution of the blood flow equations in the vascular system. In personalised applications the extra cost of identifying parameters of a distributed 1D system must be factored in though.

### Limitations and perspectives

An extension of the study is the use of more complex biventricular and four-chamber EM cardiac models [51], in order to consider the interactions among the heart chambers and thus provide more physiological simulations of the cardiac function.

Also, it is well known that cardiac preload is mainly affected by venous return. As a consequence, in order to study more accurately the feedback of vascular function (including venous return) on the cardiac function, the use of a 1D global model of the human circulation is more appropriate. Thus, a second future perspective consists in coupling the 3D EM cardiac model with closed-loop global 1D blood flow models, e.g. [52], in order to explore the effect of circulation on preload as well. We emphasise that the coupling strategy holds for a general framework consisting of multiple cardiac cavities and is prone to different discretisations and geometries for the vascular model, therefore such extensions are feasible.

Further, this study did not specifically focus on the parameterisation of the coupled model to patient-specific data. However, the personalisation of cardiovascular models is of crucial importance for their predictive role, since the vascular system has a complex network structure and shows a significant inter-individual variability. Owing to their distributed nature, 1D models of blood flow involve a larger amount of parameters that must be calibrated than lumped parameter models, related to geometric aspects, elastic properties and peripheral impedances of the network [10, 53]. This can pose serious limitations on the applicability of the use of detailed 1D vascular models in clinical applications. Nonetheless, there are numerous contributions in the scientific literature to cope with this problem, in terms of simplification of the geometry and reduction of the parameter set, together with the application of optimisation methods and robust inverse problem strategies. For example, the topological complexity of the arterial tree can be optimised by effectively reducing the number of arterial segments included in the 1D model, still preserving the key characteristics of flow and pressure waveforms [54, 55]. Common approaches also contemplate the rescaling of distal properties and vessel geometry based on allometric scales and global descriptors related to the geometry and properties of the network that are easy to measure, such as pulse wave velocity, body size, and biological age [56]. The level of detail of the network representing the vascular system should be carefully considered taking into account the specific clinical application under study.

In addition, recent parameter estimation methods based on data assimilation techniques, i.e., algorithms combining mathematical models with available measurements to improve the accuracy of model predictions and estimate patient-specific parameters, have provided promising results in cardiovascular applications [57–59].

## Conclusion

We developed a stable strategy to perform, for the first time, a coupling between a 3D EM model of the heart and a 1D blood flow model of the arterial system, based on the resolution of a saddle-point problem for the volume and pressure in the cavity. We showed robustness and accuracy of our scheme in a numerical benchmark over a range of time steps. We further demonstrated that the calibrated 3D-1D model matches qualitatively with clinical data under baseline conditions, and that known physiological responses to alterations in features of the vascular system affecting pulse wave transmission, including aortic stiffening, aortic stenosis and bifurcations, are efficiently and correctly replicated. The additional computational costs associated with the use of 1D models instead of standard 0D models in the coupled system are negligible. As a consequence, the use of this coupled 3D-1D model is beneficial for a broad spectrum of clinical applications where wave transmission effects are under study, such as aortic coarctation and pulmonary or systemic arterial hypertension.

## Appendix A: Valve dynamics models

The EM model of the heart is coupled to the arterial network via the cardiac valves. Flow through these valves is basically triggered by a positive difference pressure between the two compartments. However, several models can be considered for modelling the dynamics of these valves, ranging from simple diode-like models to more sophisticated approaches, that are able to contemplate pathological conditions such as a stenosis or regurgitation. In the literature there are three basic approaches, ranging from simple diode-like models to more complex valve models [56], simulating a simplified valve dynamics without explicitly modelling the valve leaflets. However, when it is necessary to capture more complex flow dynamics inside the ventricles that are induced by valve pathologies, it may be required to explicitly model the shape and dynamics of the leaflets. Several approaches have been proposed [60], but this is still a very open and demanding field of research. The simplest model to simulate the action of a valve corresponds to a diode associated with a resistance (characteristic of the valve), with instant opening and closing depending on the pressure gradient upstream and downstream the valve. This model has a physiological basis, since cardiac valves open and close very rapidly (in a few milliseconds). However, it does not take into account any dynamics on *Q*, and it is not possible to model pathological conditions. As a consequence, for this work we considered a more accurate model, namely a lumped parameter model of valve dynamics proposed in [39]. Its peculiarity consists in a smooth opening and closing dynamics of the valves using a simple approach. In particular, this model is derived from the Bernoulli equation, after neglecting Poiseuille-type viscous losses (proportional to *Q*), since they are small:

A1 $$\begin{aligned} \Delta P = B\, Q\, {Q} + L \, \dot{Q}. \end{aligned}$$

The Bernoulli resistance *B* is associated with the pressure difference $\Delta P$ related to convective acceleration and dynamic pressure losses caused by the diverging flow field downstream to the vena contracta, and it reads

A2 $$\begin{aligned} B = \frac{\rho }{2\, A_{\mathrm {eff}}^2}, \end{aligned}$$

where $\rho$ is the blood density (usually set equal to $1060\,\hbox {kg m}^{-3}$) and $A_\mathrm {eff}$ is an effective cross-sectional area. The blood inertance *L* is related to the pressure difference associated with blood acceleration and it is governed by the following equation:

A3 $$\begin{aligned} L = \frac{\rho \, l_{\mathrm {eff}}}{A_{\mathrm {eff}}}, \end{aligned}$$

with $l_\mathrm {eff}$ an effective length, and it is here taken equal to the diameter of $A_\mathrm {eff}$. In order to consider valve dynamics, $A_\mathrm {eff}$ is a state variable and depends on an index of valve state $\xi \in [0,1]$ ($\xi = 0$: closed valve, $\xi = 1$: open valve) such that

A4 $$\begin{aligned} A_\mathrm {eff}(t) = \big ( A_{\mathrm {eff},\max }(t) - A_{\mathrm {eff},\min }(t) \big )\xi (t) + A_{\mathrm {eff},\min }(t). \end{aligned}$$

Note that, in order to avoid division by zero in Equations (A2) and (A3), the valve state $\xi$ must be always strictly positive in practice. In order to consider pathological conditions like valve regurgitation, stenosis and varying annulus area $A_\mathrm {ann}$, the maximum $A_{\mathrm {eff},\max }$ and minimum $A_{\mathrm {eff},\min }$ effective areas can be expressed as

A5 $$\begin{aligned} \begin{aligned} A_{\mathrm {eff},\max }(t)&= M_\mathrm {st}A_\mathrm {ann}(t), \\ A_{\mathrm {eff},\min }(t)&= M_\mathrm {rg}A_\mathrm {ann}(t). \end{aligned} \end{aligned}$$

In this study, $A_\mathrm {ann}$ is considered constant. The coefficients $M_\mathrm {st}$ and $M_\mathrm {rg}$ range within [0, 1], with $M_\mathrm {rg}=0$ and $M_\mathrm {st}=1$ in case of a healthy valve, whereas $M_\mathrm {rg}>0$ indicates a regurgitant valve and $M_\mathrm {st}<1$ stands for a stenosed valve. Finally, $M_\mathrm {rg}=1$ corresponds to an absent valve (unobstructed conduit), whereas $M_\mathrm {st}=0$ stands for an atretic valve.

The rate of valve opening and closing only depends on two factors, i.e., the instantaneous pressure difference across the valve $\Delta P$ and the current state $\xi$ of the valve. In particular, it is assumed that the valve opens when $\Delta P$ exceeds a threshold $\Delta P_\mathrm {open}$, and that the rate of opening is governed by

A6 $$\begin{aligned} \frac{\mathrm {d}\xi }{\mathrm {d}t} = (1-\xi ) K_\mathrm {vo}(\Delta P - \Delta P_\mathrm {open}), \end{aligned}$$

with $K_\mathrm {vo}$ a rate coefficient for valve opening (in $\hbox {Pa}^{-1}\hbox {s}^{-1}$). On the other hand, it is assumed that the valve closes when $\Delta P$ is lower than a threshold $\Delta P_\mathrm {close}$, and that the rate of closing is governed by

A7 $$\begin{aligned} \frac{\mathrm {d}\xi }{\mathrm {d}t} = \xi \, K_\mathrm {vc}(\Delta P - \Delta P_\mathrm {close}), \end{aligned}$$

where $K_{vc}$ is a rate coefficient for valve closing (in $\hbox {Pa}^{-1}\hbox {s}^{-1}$). Model parameters are depicted in Table 3.

## Appendix B: Finite Element Formulation

### Variational Formulation

For the sake of simplicity of formulation, we neglect here the acceleration term in Eq. (3) in this section and consider at the stationary counterpart of the boundary value problem (3–19) with (14). Please refer to [34] for further detail on the full nonlinear elastodynamics problem. The boundary value problem (3–19) with (14) can be formally expressed in terms of the equations

B8 $$\begin{aligned} \langle \mathcal {A}_0({\mathbf {u}}),{\mathbf {v}}\rangle _{\Omega _0} - \langle \mathcal {F}_0({\mathbf {u}},p_c),{\mathbf {v}}\rangle _{\Omega _0}&={\mathbf {0}}, \end{aligned}$$

B9 $$\begin{aligned} \langle V_c^\mathrm {heart}({\mathbf {u}}),q\rangle _{\Omega _0} - \langle V_c^\mathrm {CS}(p_c), q\rangle _{\Omega _0}&= 0, \end{aligned}$$

which is valid for all vector fields ${\mathbf {v}}$ smooth enough and vanishing on the Dirichlet boundary ${\Gamma }_{0,\mathrm {D}}$, test functions *q* that are 1 for the cavity $c\in \{\mathrm {LV,RV,LA,RA}\}$ and 0 otherwise, with the duality pairing $\langle \cdot ,\cdot \rangle _{\Omega _0}$. The first term on the left hand side of the variational Eq. (B8) represents the rate of internal mechanical work and is defined as

B10 $$\begin{aligned} \langle \mathcal {A}_0({\mathbf {u}}) , {\mathbf {v}}\rangle _{\Omega _0} :=\int _{\Omega _0} {\mathbf {S}}({\mathbf {u}}) : \varvec{\Sigma }({\mathbf {u}},{\mathbf {v}}) \,\mathrm {d}{\mathbf {X}}, \end{aligned}$$

where ${\mathbf {S}}$ denotes the second Piola–Kirchhoff stress tensor, see (4), and $\varvec{\Sigma }({\mathbf {u}},{\mathbf {v}})$ is the directional derivative of the Green–Lagrange strain tensor, see [1, 61]. The second term on the left hand side denotes the weak form of the contribution of pressure loads (B8) and is computed using (19)

B11 $$\begin{aligned} \langle \mathcal {F}_0({\mathbf {u}},p_c),{\mathbf {v}}\rangle _{\Omega _0} = -p_c \int \limits _{\Gamma _{0,\mathrm {N}}}J\,{\mathbf {F}}^{-\top }({\mathbf {u}})\,{{\mathbf {n}}^{\mathrm {out}}_0}\cdot {\mathbf {v}}\, \,\mathrm {d}s_{{\mathbf {X}}}. \end{aligned}$$

The first term of the coupling equation (B9) is obtained from (15) using Nanson’s formula and ${\mathbf {x}}= {\mathbf {X}}+ {\mathbf {u}}$ by

B12 $$\begin{aligned} \langle V_c^\mathrm {heart}({\mathbf {u}}), q\rangle _{\Omega _0} = \frac{1}{3} \int \limits _{\Gamma _{0, \mathrm {N}}} ({\mathbf {X}}+ {\mathbf {u}}) \cdot J {\mathbf {F}}^{-\top } {{\mathbf {n}}^{\mathrm {out}}_0}\, q \,\mathrm {d}s_{{\mathbf {X}}}. \end{aligned}$$

The second term of (B9) is computed using the 1D blood flow model, see 2.2.1, for $c\in \{\mathrm {LV,RV,LA,RA}\}$.

**Table 3:** Input parameters for the valve dynamics model. Adjusted to match patient-specific data

Parameter	Value	Unit	Description
*Aortic Valve*			
$K_\mathrm {vo}$	2	$\hbox {Pa}^{-1}\hbox {s}^{-1}$	Rate coefficient for valve opening
$\Delta P_\mathrm {open}$	0	Pa	Threshold pressure difference for valve opening
$K_\mathrm {vc}$	1.2	$\hbox {Pa}^{-1}\hbox {s}^{-1}$	Rate coefficient for valve closing
$\Delta P_\mathrm {close}$	0	Pa	Threshold pressure difference for valve closing
$M_\mathrm {st}$	1	[-]	Valve stenosis coefficient
$M_\mathrm {rg}$	0	[-]	Valve regurgitation coefficient
*Mitral Valve*			
$K_\mathrm {vo}$	0.2	$\hbox {Pa}^{-1}\hbox {s}^{-1}$	Rate coefficient for valve opening
$\Delta P_\mathrm {open}$	0	Pa	Threshold pressure difference for valve opening
$K_\mathrm {vc}$	0.2	$\hbox {Pa}^{-1}\hbox {s}^{-1}$	Rate coefficient for valve closing
$\Delta P_\mathrm {close}$	0	Pa	Threshold pressure difference for valve closing
$M_\mathrm {st}$	1	[-]	Valve stenosis coefficient
$M_\mathrm {rg}$	0	[-]	Valve regurgitation coefficient

### Consistent Linearisation

A Newton–Raphson scheme is employed to solve the nonlinear variational equations (B8)–(B9), with a finite element approach see [62]. Let us consider a nonlinear and continuously differentiable operator $F:X\rightarrow Y$. Then, a solution to $F(x)=0$ can be approximated by

$$\begin{aligned}&x^{k+1} = x^{k} + \Delta x, \\&\quad \left. \frac{\partial F}{\partial x}\right| _{x = x^k} \Delta x = -F(x^k), \end{aligned}$$

which is evaluated until convergence. In our approach, we assume $X = \left[ H^1(\Omega _0,\Gamma _{0,\mathrm {D}})\right] ^3 \times \mathbb R$, $Y = \mathbb {R}^2$, $\Delta x = {(\Delta {\mathbf {u}}, \Delta p_c)}^\top$, $x^k = {({\mathbf {u}}^k, p_c^k)}^\top$, and $F = {(R_{{\mathbf {u}}},R_\mathrm {p})}^\top$. Thus, we obtain the following linearised saddle-point problem for each $({\mathbf {u}}^k, p_c^k) \in \left[ H^1(\Omega _0,\Gamma _{0,\mathrm {D}})\right] ^3 \times \mathbb R$, find $(\Delta {\mathbf {u}}, \Delta p_c) \in \left[ H^1_0(\Omega _0)\right] ^3 \times \mathbb R$ such that

B13 $$\begin{aligned} \begin{aligned} \displaystyle&\langle \Delta {\mathbf {u}},A_0'({\mathbf {u}}^k)\, {\mathbf {v}}\rangle _{\Omega _0} + \langle \Delta {\mathbf {u}},\mathcal {F}_0'({\mathbf {u}}^k, p_c^k)\,{\mathbf {v}}\rangle _{\Omega _0} \\&\quad + \langle \Delta p_c,\mathcal {F}_0'({\mathbf {u}}^k, p_c^k)\,{\mathbf {v}}\rangle _{\Omega _0}= -\langle R_{{\mathbf {u}}}({\mathbf {u}}^k, p_c^k), {\mathbf {v}}\rangle _{\Omega _0}, \end{aligned} \end{aligned}$$

B14 $$\begin{aligned} \begin{aligned} \displaystyle&\langle \Delta {\mathbf {u}},V_c^\mathrm {heart}({\mathbf {u}}^k)\, q\rangle _{\Omega _0} - \langle \Delta p_c, V_c^\mathrm {CS}(p_c^k)\, q \rangle _{\Omega _0} \\& = -\langle R_\mathrm {p}({\mathbf {u}}^k, p_c^k), q\rangle _{\Omega _0}, \end{aligned} \end{aligned}$$

with the updates

B15 $$\begin{aligned} {\mathbf {u}}^{k+1}&={\mathbf {u}}^k+\Delta {\mathbf {u}}, \end{aligned}$$

B16 $$\begin{aligned} p_c^{k+1}&=p_c^k+\Delta p_c. \end{aligned}$$

The Gâteaux derivative of (B8) with respect to the displacement change update $\Delta {\mathbf {u}}$ yields the first term in (B13)

B17 $$\begin{aligned} \begin{aligned}&\langle \Delta {\mathbf {u}},A_0'({\mathbf {u}}^k)\, {\mathbf {v}}\rangle _{\Omega _0}\\&\quad := \left. D_{\Delta {\mathbf {u}}} \langle \mathcal {A}_0({\mathbf {u}}), {\mathbf {v}}\rangle _{\Omega _0}\right| _{{\mathbf {u}}={\mathbf {u}}^k} \\&\quad \, = \int \limits _{\Omega _0} {\mathbf {S}}_k: {\Sigma }(\Delta {\mathbf {u}}, {\mathbf {v}})\,\mathrm {d}{\mathbf {X}}\\&\qquad \, + \int \limits _{\Omega _0} \Sigma ({\mathbf {u}}^k, \Delta {\mathbf {u}}) : \mathbb {C}_k : \Sigma ({\mathbf {u}}^k, {\mathbf {v}})\,\mathrm {d}{\mathbf {X}}, \end{aligned} \end{aligned}$$

and the second term in (B13), that is given by

B18 $$\begin{aligned} \begin{aligned} \displaystyle&\langle \Delta {\mathbf {u}},\mathcal {F}_0'({\mathbf {u}}^k, p_c^k)\,{\mathbf {v}}\rangle _{\Omega _0} \\&\quad :=\left. D_{\Delta {\mathbf {u}}} \langle \mathcal {F}_0({\mathbf {u}},p_c), {\mathbf {v}}\rangle _{\Omega _0}\right| _{{\mathbf {u}} ={\mathbf {u}}^k, p_c = p_c^k}\\&\quad \, = p^k_c \int _{\Gamma _{0,\mathrm {N}}}J_k{\mathbf {F}}_k^{-\top }{\text {Grad}}^\top \!\Delta {\mathbf {u}}\, {\mathbf {F}}_k^{-\top }{{\mathbf {n}}^{\mathrm {out}}_0}\cdot {\mathbf {v}}\, \,\mathrm {d}s_{{\mathbf {X}}}\\&\quad \, \ -p^k_c \int _{\Gamma _{0,\mathrm {N}}}J_k({\mathbf {F}}_k^{-\top }:{\text {Grad}}\Delta {\mathbf {u}})\, {\mathbf {F}}_k^{-\top }{{\mathbf {n}}^{\mathrm {out}}_0}\cdot {\mathbf {v}}\, \,\mathrm {d}s_{{\mathbf {X}}}, \end{aligned} \end{aligned}$$

where we have defined, for the sake of simplicity:

$$\begin{aligned} {\mathbf {F}}_k&:= {\mathbf {F}}({\mathbf {u}}^k),\quad \, J_k := \det ({\mathbf {F}}^k)\\ {\mathbf {S}}_k&:= \left. {\mathbf {S}}\right| _{{\mathbf {u}}={\mathbf {u}}^k},\ \mathbb {C}_k:= \left. \mathbb {C}\right| _{{\mathbf {u}}={\mathbf {u}}^k}. \end{aligned}$$

The third term in (B13) is defined using the Gâteaux derivative of (B8) with respect to the pressure change update $\Delta p_c$:

B19 $$\begin{aligned} \begin{aligned} \displaystyle&\langle \Delta p_c,\mathcal {F}_0'({\mathbf {u}}^k, p_c^k)\,{\mathbf {v}}\rangle _{\Omega _0} \\&\qquad := \left. D_{\Delta p_c} \langle \mathcal {F}_0({\mathbf {u}},p_c), {\mathbf {v}}\rangle _{\Omega _0}\right| _{{\mathbf {u}} ={\mathbf {u}}^k, p_c = p_c^k} \\&\qquad \, = -\Delta p_c \int _{\Gamma _{0,\mathrm {N}}}J_k\,{\mathbf {F}}_k^{-\top }\,{{\mathbf {n}}^{\mathrm {out}}_0}\cdot {\mathbf {v}}\, \,\mathrm {d}s_{{\mathbf {X}}}. \end{aligned} \end{aligned}$$

The right hand side of (B13) depends on the residual $R_{{\mathbf {u}}}$, and it is defined as

B20 $$\begin{aligned} \begin{aligned} \langle R_{{\mathbf {u}}}({\mathbf {u}}^k, p_c^k), {\mathbf {v}}\rangle _{\Omega _0} :=&\langle A_0({\mathbf {u}}^k),{\mathbf {v}}\rangle _{\Omega _0} \\&\ -\langle {\mathbf {\mathcal {F}}}_0({\mathbf {u}}^k, p_c^k),{\mathbf {v}}\rangle _{\Omega _0}. \end{aligned} \end{aligned}$$

From (B12), using the known relations [61],

$$\begin{aligned} \frac{\partial J}{\partial {\mathbf {F}}} : {\text {Grad}}\Delta {\mathbf {u}}&= J {\mathbf {F}}^{-\top } : {\text {Grad}}\Delta {\mathbf {u}}\\ \frac{\partial {\mathbf {F}}^{-\top }}{\partial {\mathbf {F}}} : {\text {Grad}}\Delta {\mathbf {u}}&= -{\mathbf {F}}^{-\top }{({\text {Grad}}\Delta {\mathbf {u}})}^\top {\mathbf {F}}^{-\top } \end{aligned}$$

we can define the first term of (B14) as the Gâteaux derivative with respect to the update $\Delta {\mathbf {u}}$

B21 $$\begin{aligned} \displaystyle&\langle \Delta {\mathbf {u}},V_c^\mathrm {heart}({\mathbf {u}}^k)\, q\rangle _{\Omega _0}\end{aligned}$$

B22 $$\begin{aligned}&\quad := \left. D_{\Delta {\mathbf {u}}} \langle V_c^\mathrm {heart}({\mathbf {u}}),q\rangle _{\Omega _0} \right| _{{\mathbf {u}}={\mathbf {u}}^k} \nonumber \\&\quad \, = D_{\Delta {\mathbf {u}}} \frac{1}{3}\int _{\Gamma _{0,\mathrm {N}}} \left( {\mathbf {X}}+ {\mathbf {u}}^k\right) \cdot J_k {\mathbf {F}}_k^{-\top } {{\mathbf {n}}^{\mathrm {out}}_0}\,q \,\mathrm {d}s_{{\mathbf {X}}}\nonumber \\&\quad \, = \frac{1}{3} \int _{\Gamma _{0,\mathrm {N}}} J_k ({\mathbf {F}}_k^{-\top } : {\text {Grad}}\Delta {\mathbf {u}}) {\mathbf {x}}\cdot {\mathbf {F}}_k^{-\top } {{\mathbf {n}}^{\mathrm {out}}_0}\,q \,\mathrm {d}s_{{\mathbf {X}}}\nonumber \\&\quad \ - \frac{1}{3} \int _{\Gamma _{0,\mathrm {N}}} J_k {\mathbf {x}}\cdot {\mathbf {F}}_k^{-\top }{({\text {Grad}}\Delta {\mathbf {u}})}^\top {\mathbf {F}}_k^{-\top } {{\mathbf {n}}^{\mathrm {out}}_0}\,q \,\mathrm {d}s_{{\mathbf {X}}}\nonumber \\&\quad \ + \frac{1}{3} \int _{\Gamma _{0,\mathrm {N}}} J_k \Delta {\mathbf {u}}\cdot {\mathbf {F}}_k^{-\top }{{\mathbf {n}}^{\mathrm {out}}_0}\,q \,\mathrm {d}s_{{\mathbf {X}}}, \end{aligned}$$

where *q* is a test function with value 1 for the surface $\Gamma _{0,c}$ of cavity *c*, and 0 otherwise.

The second term of (B14) is defined as the numerical derivative

B23 $$\begin{aligned} \begin{aligned} \displaystyle&\langle \Delta p_c, V_c^\mathrm {CS}(p_c^k)\, q \rangle _{\Omega _0}\\&\quad := \left. D_{\Delta p_c} \langle V_c^\mathrm {CS}(p_c)\, q \rangle _{\Omega _0} \right| _{p_c = p_c^k} \\&\quad \, = \frac{1}{\epsilon }\left( V_c^\mathrm {CS}(p_c^k + \epsilon ) - V_c^\mathrm {CS}(p_c^k)\right) q, \end{aligned} \end{aligned}$$

with $\epsilon =p_c^k\sqrt{\epsilon _\mathrm {m}}$ chosen according to [63, Chapter 5.7], and $\epsilon _\mathrm {m}=2^{-52}\approx 2.2*10^{-16}$ is the machine accuracy.

Finally, the right hand side of (B14), is defined based on the residual $R_\mathrm {p}$ and reads

B24 $$\begin{aligned} \begin{aligned} \langle R_\mathrm {p}({\mathbf {u}}^k, p_c^k), q\rangle _{\Omega _0} :=&\langle V_c^\mathrm {heart}({\mathbf {u}}),q\rangle _{\Omega _0} \\&\ - \langle V_c^\mathrm {CS}(p_c), q\rangle _{\Omega _0}. \end{aligned} \end{aligned}$$

Assembling of the block matrices The finite element method (FEM) relies on the definition of an admissible decomposition of the computational domain $\Omega \subset \mathbb {R}^3$ into *M* tetrahedral elements $\tau _j$ and the introduction of a conformal finite element space

$$\begin{aligned} X_h \subset H^1(\Omega _0), \ N = \text {dim}\, X_h \end{aligned}$$

of piecewise polynomial continuous basis functions $\varphi _i$. After linearising the variational problem (B13)–(B14) and considering a Galerkin finite element discretisation, we obtain a block system to be solved. The problem then reads: find $\delta \underline{u}\in \mathbb {R}^{3N}$ and $\delta \underline{p}_c\in \mathbb {R}^{N_\mathrm {cav}}$ such that

B25 $$\begin{aligned} \begin{aligned} \begin{pmatrix} ({\mathbf {A}}'-{\mathbf {M}}')(\underline{u}^k, \underline{p}_c^k) &{} {\mathbf {B}}'_\mathrm {p}(\underline{u}^k) \\ {\mathbf {B}}'_{{\mathbf {u}}}(\underline{u}^k) &{} {\mathbf {C}}'(\underline{p}_c^k) \\ \end{pmatrix} \begin{pmatrix} \delta \underline{u}\\ \delta \underline{p}_c \end{pmatrix} \\ = - \begin{pmatrix} \underline{A}(\underline{u}^k)-\underline{B}_\mathrm {p}(\underline{u}^k,\underline{p}_c^k) \\ \underline{V}_c^\mathrm {heart}(\underline{u}^k) -\underline{V}_c^\mathrm {CS}(\underline{p}_c^k) \end{pmatrix}, \end{aligned} \end{aligned}$$

$$\begin{aligned} \underline{u}^{k+1}&= \underline{u}^{k} + \delta \underline{u},\\ \underline{p}_c^{k+1}&= \underline{p}_c^k + \delta \underline{p}_c \end{aligned}$$

with the solution vectors $\underline{u}^k\in \mathbb {R}^{3N}$ and $\underline{p}_c^k\in \mathbb {R}^{N_\mathrm {cav}}$ at the *k*-th Newton step. The tangent stiffness matrix ${\mathbf {A}}'\in \mathbb {R}^{3N\times 3N}$ is computed from (B17) as follows:

B26 $$\begin{aligned} {\mathbf {A}}'(\underline{u}^k)[j,i] := \langle \varvec{\varphi }_i, \mathcal {A}_0'({\mathbf {u}}^k)\, \varvec{\varphi }_j \rangle _{\Omega _0}. \end{aligned}$$

The mass matrix ${\mathbf {M}}'\in \mathbb {R}^{3N\times 3N}$ is calculated from (B18) and reads

B27 $$\begin{aligned} {\mathbf {M}}'(\underline{u}^k, \underline{p}_c^k)[j,i] := \langle \varvec{\varphi }_i, \mathcal {F}_0'({\mathbf {u}}^k, p_c^k)\, \varvec{\varphi }_j \rangle _{\Omega _0}. \end{aligned}$$

The off-diagonal matrices ${\mathbf {B}}'_{{\mathbf {u}}}\in \mathbb {R}^{3N\times N_{\mathrm {cav}}}$ and ${\mathbf {B}}'_{\mathrm {p}}\in \mathbb {R}^{N_\mathrm {cav}\times 3N}$ in (B25) are assembled using (B22)

B28 $$\begin{aligned} \begin{aligned} {\mathbf {B}}'_{{\mathbf {u}}}(\underline{u}^k,\underline{p}_c^k)[i,j] = \langle \underline{\varphi }_j, V_c^\mathrm {heart}({\mathbf {u}}^k)\hat{\varphi _i}\rangle _{\Omega _{0}},\\ i=1,\dots ,N_\mathrm {cav} \end{aligned} \end{aligned}$$

and (B19)

B29 $$\begin{aligned} \begin{aligned} {\mathbf {B}}'_{\mathrm {p}}(\underline{u}^k,\underline{p}_c^k)[i,j] = \langle \hat{\varphi _j},\mathcal {F}_0'({\mathbf {u}}^k, p_c^k) \varvec{\varphi }_i\rangle _{\Omega _0}, \\ j=1,\dots ,N_\mathrm {cav}, \end{aligned} \end{aligned}$$

where the constant shape functions have value $\hat{\varphi _j}=1$ if $\tau _j\in \Gamma _{0,c}$ and $\hat{\varphi _j}=0$ if $\tau _j\notin \Gamma _{0,c}$ for $c\in \{\mathrm {LV,RV,LA,RA}\}$.

Considering the approach described in [64, Sect. 4.2], this assembling procedure can be simplified for closed cavities such that

$$\begin{aligned} {\mathbf {B}}'_{\mathrm {p}}(\underline{u}^k,\underline{p}_c^k) = {\left[ {\mathbf {B}}'_{{\mathbf {u}}}(\underline{u}^k,\underline{p}_c^k)\right] }^\top . \end{aligned}$$

Then, the vascular compliance matrix ${\mathbf {C}}'(\underline{p}_c^k)\in \mathbb {R}^{N_\mathrm {cav}\times N_\mathrm {cav}}$ is computed from (B23) as

B30 $$\begin{aligned} \begin{aligned} {\mathbf {C}}'(\underline{p}_c^k)[i,j]= \langle \hat{\varphi _j}, V_c^\mathrm {CS}(p_c^k)\, \hat{\varphi _i}\rangle _{\Omega _0}, \\ i,j=1,\dots ,N_\mathrm {cav}, \end{aligned} \end{aligned}$$

with the constant shape function $\hat{\varphi _j}=1$ if $\tau _j\in \Gamma _{0,c}$ and $\hat{\varphi _j}=0$ if $\tau _j\notin \Gamma _{0,c}$ for $c\in \{\mathrm {lv,rv,la,ra}\}$, leading to a diagonal matrix. The terms on the right hand side $\underline{A}\in \mathbb {R}^{3N}$, $\underline{B}_\mathrm {p}\in \mathbb {R}^{3N}$ are constructed using (B20) and read

B31 $$\begin{aligned} \underline{A}(\underline{u}^k)[i] := \langle \mathcal {A}_0({\mathbf {u}}^k),\varvec{\varphi }_i \rangle _{\Omega _0} \end{aligned}$$

and

B32 $$\begin{aligned} \underline{B}_\mathrm {p}(\underline{u}^k, \underline{p}_c^k)[i] := \langle \mathcal {F}_0({\mathbf {u}}^k, p_c^k),\varvec{\varphi }_i \rangle _{\Omega _0}, \end{aligned}$$

see also [1, 61]. Finally, the terms $\underline{V}_c^\mathrm {heart}\in \mathbb {R}^{N_\mathrm {cav}}$ and $\underline{V}_c^\mathrm {CS}\in \mathbb {R}^{N_\mathrm {cav}}$ in lower right hand side in (B25) are assembled from (B24) and are given by:

B33 $$\begin{aligned} \underline{V}_c^\mathrm {heart}(\underline{u}^k)[i] =\langle V_c^\mathrm {heart}({\mathbf {u}}),\hat{\varphi _i}\rangle _{\Omega _0},\ i=1,\dots ,N_\mathrm {cav}, \end{aligned}$$

and

B34 $$\begin{aligned} \underline{V}_c^\mathrm {CS}(\underline{p}_c^k)[i] =\langle V_c^\mathrm {CS}(p_c), \hat{\varphi _i}\rangle _{\Omega _0},\ i=1,\dots ,N_\mathrm {cav}. \end{aligned}$$

## Appendix C: Direct Schur Complement Solver for a Small Number of Constraints

Let us consider the block system ${\mathbf {K}}\in \mathbb {R}^{n\times n}$, ${\mathbf {C}}\in \mathbb {R}^{m\times m}$

$$\begin{aligned} \begin{pmatrix} {\mathbf {K}}&{}{\mathbf {A}}\\ {\mathbf {B}}&{}{\mathbf {C}} \end{pmatrix} \begin{pmatrix} \underline{u}\\ \underline{p} \end{pmatrix}=- \begin{pmatrix} \underline{f}\\ \underline{g} \end{pmatrix} \end{aligned}$$

with

We can write the Schur complement system as

With

C35

we obtain

C36 $$\begin{aligned} ({\mathbf {B}}{\mathbf {S}}-{\mathbf {C}})\underline{p}&=\underline{g}-{\mathbf {B}}\underline{r}\nonumber \\ \underline{u}&=\underline{r}-{\mathbf {S}}\underline{p}. \end{aligned}$$

The realisation of (C36) involves $m+1$ solves and the inversion of an $m\times m$ matrix. As *m* is generally small, this can be done symbolically.

$$\begin{aligned} {[{\mathbf {B}}{\mathbf {S}}]}_{ij}=\underline{b}_i\cdot \underline{s}_j,\ \text{ for }\ i,j=1,\dots ,m. \end{aligned}$$
